# Controllable secretion of multilayer vesicles driven by microbial polymer accumulation

**DOI:** 10.1038/s41598-022-07218-z

**Published:** 2022-03-01

**Authors:** Sangho Koh, Michio Sato, Kota Yamashina, Yuki Usukura, Masanori Toyofuku, Nobuhiko Nomura, Seiichi Taguchi

**Affiliations:** 1 Graduate School of Science, Technology and Innovation, Kobe University, 1-1 Rokkodai-cho, Nada, Kobe, 657-8501 Japan; 2 Department of Chemistry for Life Sciences and Agriculture, Faculty of Life Sciences and Agriculture, Tokyo University of Agriculture, 1-1-1 Sakuragaoka, Setagaya, Tokyo 156-8502 Japan; 3 School of Agriculture, Meiji University, 1-1-1 Higashimita, Tama, Kawasaki, 214-8571 Japan; 4 Graduate School of Life and Environmental Sciences, University of Tsukuba, Tsukuba, Ibaraki 305-8572 Japan; 5 Suntory Rising Stars Encouragement Program in Life Sciences (SunRiSE), 8-1-1 Seikadai, Soraku, Kyoto, 619-0284 Japan; 6 Microbiology Research Center for Sustainability, University of Tsukuba, Ibaraki, 305-8572 Japan

**Keywords:** Environmental biotechnology, Industrial microbiology

## Abstract

Membrane vesicles (MVs) are formed in various microorganisms triggered by physiological and environmental phenomena. In this study, we have discovered that the biogenesis of MV took place in the recombinant cell of *Escherichia coli* BW25113 strain that intracellularly accumulates microbial polyester, polyhydroxybutyrate (PHB). This discovery was achieved as a trigger of foam formation during the microbial PHB fermentation. The purified MVs were existed as a mixture of outer MVs and outer/inner MVs, revealed by transmission electron microscopy. It should be noted that there was a good correlation between MV formation and PHB production level that can be finely controlled by varying glucose concentrations, suggesting the causal relationship in both supramolecules artificially produced in the microbial platform. Notably, the controllable secretion of MV was governed spatiotemporally through the morphological change of the *E. coli* cells caused by the PHB intracellular accumulation. Based on a hypothesis of PHB internal-pressure dependent envelope-disorder induced MV biogenesis, here we propose a new Polymer Intracellular Accumulation-triggered system for MV Production (designated “PIA-MVP”) with presenting a mechanistic model for MV biogenesis. The PIA-MVP is a promising microbial platform that will provides us with a significance for further study focusing on biopolymer capsulation and cross-membrane transportation for different application purposes.

## Introduction

Poly(3-hydroxyalkanoate) (PHA) is intracellularly accumulated as a carbon and energy storage polymer in various microorganisms under nutrient imbalance conditions^1^. PHA accumulation is affected by the availability of nutritional resources and this knowledge has been used to establish culture conditions favoring high productivities. Also, a link between PHA accumulation and stress tolerance has been observed in some bacteria, showing that PHA has more biological roles than merely a carbon and energy storage molecule^2^. Thus, there are many suggestions that PHA metabolism acts as a regulatory mechanism optimizing the carbon and energy flow in the natural bacterial cell^2^.

Nowadays, many attempts for PHA overproduction have been extensively carried out in the recombinant bacterial platforms in order to supply this biodegradable polymer to the urgent demands applicable for solving a microplastic problem especially in marine^3,4^. Since the biodegradable PHA can be synthesized from renewable carbon sources, this carbon neutral polymer would be an ideal item to establish sustainable and circular bioeconomy. For this purpose, *Escherichia coli*, a PHA non-producer, is most frequently used as a host cell for metabolically engineering strains and their fermentation for optimizing PHA production. So far, PHA has been considered to be the primary target product. However, recently, it was reported that PHA functions as a carbon reservoir for improving the yield of secondary metabolites such as antibiotic, when carbon sources are insufficient^5^. This suggests that PHA would potentially become any by-player(s) for production of the value-added targets of interest.

In this study, unexpectedly, we for the first time found the biogenesis of membrane vesicle (MV) in *E. coli* by intracellular accumulation of poly(3-hydroxybutyrate) (PHB), a most-studied PHA. A discovery of MV biogenesis has been motivated by the reproducible appearance of a lot of foam during the aerobic cultivation of the PHB producing recombinant strain. Most of the PHA researchers probably have overlooked this phenomenon as being used to be not special. In such a sense, the appearance of foam could be a good monitoring indicator for MV biogenesis. Conversely, MV biogenesis raises the fundamental issue, physiological effect occurred by intracellular accumulation of PHB in *E. coli*.

Here we have demonstrated the **P**olymer **I**ntracellular **A**ccumulation-triggered system for **MV** **P**roduction (designated “PIA-MVP”) with several experimental findings, microscopic observations, and detailed component analysis for fractionated MVs. Notably, the secretion of MVs was well proportioned to the PHB production level that can be finely controlled by the concentration of glucose externally added as a carbon source. This causal relationship between both highly-organized supramolecules was found to be spatiotemporally modulated through the morphological change of the *E. coli* cells. Thus, we have proposed a hypothetical model of PIA-MVP based on the data gained here together with previous reports. The newly identified PIA-MVP will have a significance on both research fields of the popular supramolecules, PHB and MV.

## Results and discussion

### Discovery of the biogenesis of membrane vesicle in *E. coli* upon PHB production

**Figure 1:**
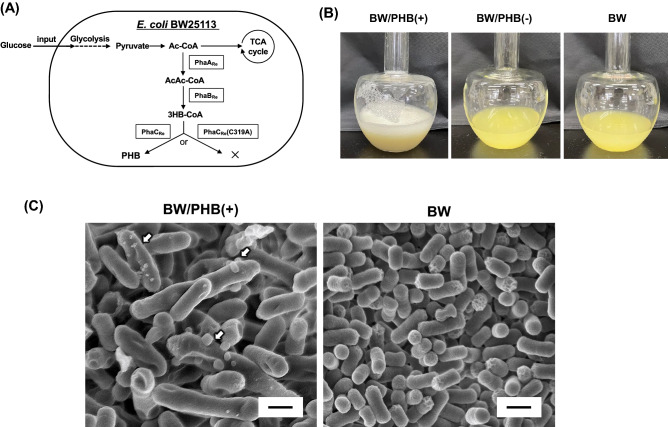
The primary findings of MV biogenesis in *E. coli* producing PHB. (**A**) Schematic illustration of a metabolic pathway for production of PHB. Acetyl-CoA (Ac-CoA) supplied from glucose via glycolysis is converted into acetoacetyl-CoA (AcAc-CoA) through function of heterologously expressed PhaA_Re_. AcAc-CoA is converted into 3-hydroxybutyrate-CoA (3HB-CoA) through function of PhaB_Re_. 3HB-CoA is utilized as a monomer for the polymerization by PhaC_Re_. When the inactive mutant PhaC(C319A) is replaced to PhaC_Re_, the production of PHB is not observed. (**B**) The foam observed during cultivation. (**C**) SEM images of the cell surface of three recombinant constructs: BW/PHB (+), BW/PHB (−) and BW. Black arrowheads denote MV-like particles budded from the cell surface. Scale bars represent 1 μm.

Figure 1A shows the synthetic pathway for production of PHB in *Escherichia coli*. PHB is synthesized by sequential enzyme reactions of 3HB-CoA-precusor supplying steps catalyzed by β-ketothiolase (PhaA_Re_) and NADPH-dependent acetoacetyl-CoA reductase (PhaB_Re_), and polymerizing step catalyzed by PHB synthase (PhaC_Re_). The finding of MV biogenesis in our microbial system has been motivated by the reproducible appearance of a lot of bubbles during the aerobic cultivation of the PHB-producing recombinant strain of *E. coli*. Namely, when the recombinant strain carrying the PHB synthetic pathway, BW/PHB (+) (BW25113 harboring pGEM-*phaC*_Re_*AB*)^6^ was cultivated, we observed a lot of foam in the Sakaguchi flask (Fig. 1B). As shown in Table 1, the cellular content of PHB was 60 ± 4 wt% in the LB medium containing 2% glucose. On the other hand, we confirmed that this phenomenon was not observed for BW/PHB (−) harboring the inactivated-PHB synthase gene [*phaC*_Re_(C319A)]^6^ and the parent strain *E. coli* BW25113 (BW) with no plasmid (Fig. 1B). Therefore, the phenomenon was specific for PHB-producing strain. This prompted us to further investigate the foam by several analyses. As a result, it was suggested that the foam contains a lipid at least that was changed to be dark, when the sample was exposed to a fixation-liquid (osmium tetroxide) for transmission electron microscopic observation (unpublished data). Then, we carried out the microscopic observation of the culture samples containing BW/PHB (+) and BW at 48 h culture. As the result, several particles (approximately 100 nm scale) were budded on the surface of single cell of BW/PHB (+) using scanning electron microscopy (SEM) (Fig. 1C). Based on these morphological observations, we postulated that such a kind of budded particles would be a membrane vesicle (MV) referring the previous papers^7^.

**Table 1:** Summary of dry-cell weight, PHB contents and MV production of each *E. coli* strain.

Strain	Dry-cell weight (g/L)	PHB contents (wt%)	MV production (FM1-43 FX) (arbitrary)
BW/PHB (+)	12.3 ± 3.6	60 ± 4	2634 ± 556
BW/PHB (−)	1.3 ± 0.1	ND	20 ± 10
BW	1.2 ± 0.1	ND	36 ± 12

Cells were cultivated in LB medium containing 2% (w/v) glucose at 30 °C for 48 h. The results are the averages ± standard deviations from three independent experiments.

**Figure 2:**
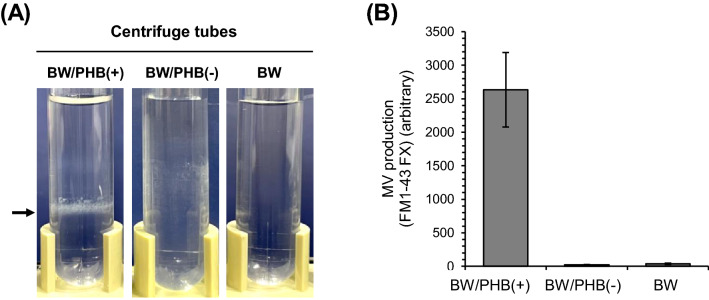
Comparison in the amount of the MV-fractions isolated from culture supernatant of BW/PHB (+), BW/PHB (−) and BW. (**A**) MV purified by density gradient-ultracentrifugation. The black arrow indicates MV fraction for BW/PHB (+). (**B**) Comparison of MV production. The data are shown as the mean ± standard deviation from three independent experiments.

**Figure 3:**
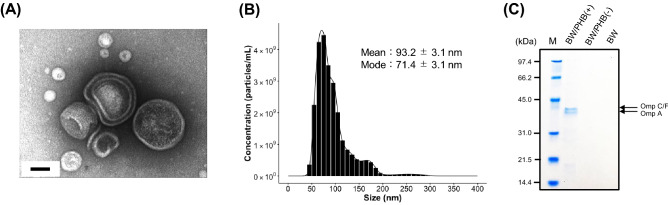
Microscopic and biochemical characterization of the MVs. (**A**) TEM images of MV fractions of BW/PHB (+). Scale bar represents 100 nm. (**B**) The particle size distribution was analyzed by NanoSight tracking analysis. (**C**) Protein profiles of MV preparations from BW/PHB (+), BW/PHB (−) and BW. Purified MV samples were analyzed by SDS-PAGE using 12.0% acrylamide gel and stained with CBB. Estimated proteins of the main bands are shown on the right. Lane M represents the marker.

In the next, we attempted to isolate and purify the MV-like particles by using iodixanol density-gradient ultracentrifugation according to the following scheme^8^. After density-gradient ultracentrifugation, the particles-contained fraction was analyzed based on the staining by using a fluorescent FM1-43 FX (data not shown). Figure 2A shows the comparison in the quantity of particles-contained band in the centrifuge tubes (BW/PHB (+), BW/PHB (−) and BW). The extraction of MVs from the sample of BW/PHB (+) was also monitored based on fluorescent intensity of FM1-43 FX (Fig. 2B). Transmission electron microscopic (TEM) analysis also revealed the existence of MVs in the collected fraction (Fig. 3A). The TEM image shows the presence of a mixture of single-layered and multilayered MVs. Next, the diameter of MVs was measured by Nanosight tracking analysis, and the size distribution was plotted (Fig. 3B). The average particle size of MVs from the BW/PHB (+) cells was 93.2 ± 3.1 nm, consistent with that observed in the parent strain of *E. coli* BW25113, as seen in the previous study^9,10^. In addition, predicted outer membrane protein bands approximately at 37 kDa (OmpF/C, and OmpA) which provide an index of MV generation^9^, were detected by SDS-PAGE analysis (Fig. 3C). These results suggested that the BW/PHB (+) cells released MVs into the culture medium. Finally, we have concluded the biogenesis of MV.

The PHB intracellular accumulation-triggered MV biogenesis, PIA-MVP, is a complete new from both research fields, PHB and MV. So far, no one has recognized this unexpected PIA-MVP during cultivation of the recombinant *E. coli* producing PHB that so many researchers usually conduct. From the side of a long research history on MV biogenesis, PIA-MVP is a different principle and approach. In order to address the molecular mechanism of PIA-MVP, we have focused on spaciotemporal relationship of both supramolecules through morphological change of the cells as follows.

### Time courses of cell growth, intercellular accumulation of PHB and MV release

**Figure 4:**
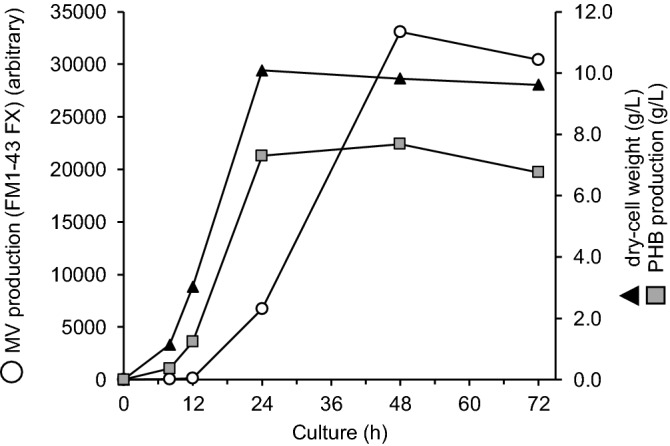
Time course profiles of MV production, PHB production and cell growth by BW/PHB (+). BW/PHB (+) was cultivated in LB medium containing 2.0% glucose at 30 °C for 72 h.

The PIA-MVP has been demonstrated in the recombinant PHB-producing *E. coli* by several biochemical analyses, as described above. Then, to better understand the relationship of both supramolecules, we conducted the time courses for production of PHB and MV as well as cell growth, using the BW/PHB (+) during the cultivation times (up to 72 h). As shown in Fig. 4, the PHB production was associated with cell growth. The released MVs were quantified with a fluorescent FM1-43 FX according to a previously described method with minor modifications^11^. It seemed that MVs began to be released at a little bit of delay (24–48 h) after the PHB-accumulation was reached the saturation level. This suggests a tight relationship between MVs secretion and PHB-accumulation based on the fact that the release of MV was initiated in response to the PHB accumulation. This should be an important finding in considering the PIA-MVP established in this study.

### Relationship between PHB accumulation level and MV production level

**Figure 5:**
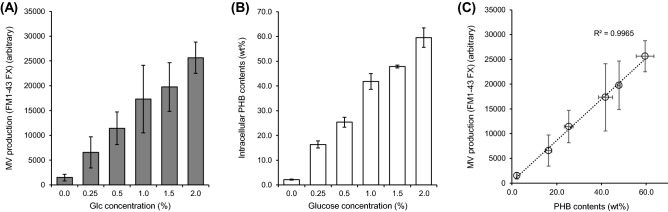
Relationship between MV production and intracellular PHB contents. (**A**) PHB production and (**B**) MV production of BW/PHB (+) under five different glucose concentrations (0–2.0%) for 48 h cultivation. (**C**) The relationship between the PHB production level and MV production level. The MV production clearly increased with increasing intracellular PHB production. The data are shown as the mean ± standard deviation from three independent experiments.

To further investigate the relationship between both supramolecules, we next quantitatively analyzed the intracellularly accumulated content of PHB and the amount of released MVs. First, we attempted to control the PHB accumulation level by varying the glucose concentration as a carbon source. Figure 5A shows a good glucose concentration-dependent production of PHB. It means that the PHB production level can be fine-tuned by controlling the glucose concentration. Also, the amount of the released MVs was in good proportion to the PHB production level as shown in Fig. 5B. Surprisingly, we obtained a linear relationship between PHB production level and MV production level with an extremely high correlation coefficient of R^2^ = 0.99625, as shown in Fig. 5C. Accordingly, it should be noted that the MV secretion can be fine-tuned by regulating the glucose concentration. This glucose-controllable approach would be very effective for investigating the mechanism PIA-MVP and wide-range of applications, as mentioned below.

### Morphological change of the cells associated with PHB production

**Figure 6:**
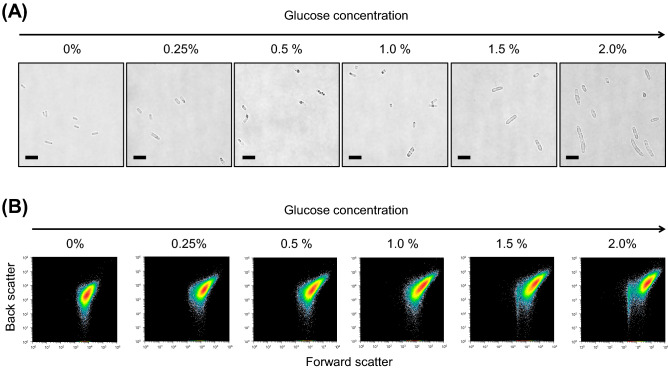
Morphological change of the BW/PHB (+) cells by varying glucose concentration. Cells were cultivated under five different glucose concentrations (0% to 2.0%) at 30 °C for 48 h cultivation. (**A**) Microscopic images of the BW/PHB(+) cells. Scale bars, 5 μm. (**B**) Flow cytometry to determine change in cell size. The intensity of forward scatter signals and back scatter signals are related to cell size and granularity, respectively.

In the next, we investigated the cell itself probably affected by the enhancements in PHB production and MV secretion tightly associated with the concentration of added glucose. As shown in Fig. 6A, the cell volume was increased in a proportional manner while increasing the glucose concentrations ranging from 0 to 2%. The enlargement of single cells can be probably accounted for by the internal pressure caused by the intracellular accumulation of PHB. Such a glucose concentration dependency was not observed for PHB non-producing strain at all (Supplementary Fig. S1). Therefore, it can be concluded that glucose-dependent morphological change was specifically occurred in the BW/PHB (+).

In addition, the flow-cytometry allowed us to analyze a great number of cells independently. The distribution of cell size can be statistically evaluated by two parameters, signal intensities of forward scatter and back scatter. Forward scatter and back scatter correspond to the cell size and granularity, respectively. The pattern showing upper-right shift means the increase in the cell size. As shown in Fig. 6B, both forward scatter and back scatter signals were increased with increasing the glucose concentration, indicated that the relative cell size was increased depending on the glucose concentration, consistent with as mentioned above. The dose-dependent enlargement of the cells reminds us to reconfirm the frequency of blebbed forms of MV appeared on the cell surface. Therefore, these three physiological parameters were finely governed by glucose concentration. Notably, the glucose concentration-dependent PHB production is a key trigger for the cell enlargement and MV biogenesis. In short, it can be concluded that PIA-MVP spatiotemporally takes place via cellular morphological changes. This should be a crucial finding for establishing the molecular mechanism of PIA-MVP.

### A hypothetical model for PIA-MVP

In case of Gram-negative bacteria like *E. coli*, MV biogenesis is promoted through several pathways such as imbalance of peptidoglycan and membrane synthesis^12^. Especially, an envelope stress in the membrane have been established well for MV production^13–15^. The envelope stress is caused by disorder of the linkage networking between three structured biomacromolecules, outer membrane-peptidoglycan-inner membrane. Accordingly, many spontaneous and artificial mutations closely related to hypervesiculation are noticeably distributed in the cell^9,10^.

**Figure 7:**
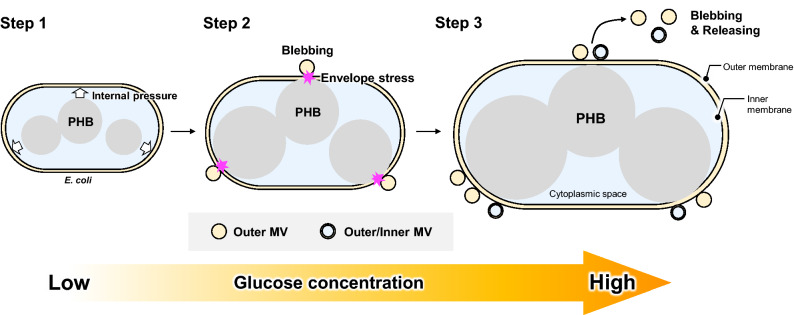
A hypothetical model for the Polymer Intracellular Accumulation-triggered system for MV Production (PIA-MVP). A hypothetical model for PIA-MVP is made based on the following these three steps. (Step 1) PIA-MVP would take place via the internal pressure caused by PHB accumulation. (Step 2) the internal pressure may give a physical perturbation to the linkage network formed between three structurered biomacromolecules, outer membrane-peptidoglycan-inner membrane. Consequently, a disordered state would be occurred in the periplasmic space as an envelope stress, causing MV biogenesis. (Step 3) single-layer MVs (Outer MVs, OMVs) and multilayer MVs (Outer/Inner MVs, OIMVs) frequently occur. The illustration was drawn by the drawing tool in Microsoft PowerPoint ver. 16.56 (Microsoft).

On the other hand, PIA-MVP has provided us with a different principle in which a trigger for MV formation is PHB accumulation itself. Namely, there is a linear relationship between the amount of released MVs and PHB production level, associated with accompanying the morphological change of the cells. Thus, it might be considered that the change in cell volume correlates well with the internal pressure, as it were an envelope stress, induced by PHB accumulation (Fig. 7). As illustrated in Fig. 7, the secretion level of MVs can be controlled by the concentration of glucose externally added.

It is often argued that the formation of single-layer MV (Outer MV, OMV) and multilayer MV (Outer/Inner MV, OIMV) takes place depending on the given physiological situations in Gram-negative bacteria^12^. As mentioned above (Fig. 3A), in our PIA-MVP system, there are a mixture of OMVs and OIMVs in the culture supernatant. Taking the advantage of the OIMVs formation would be utilized as a cargo for encapsuling intracellularly polymerized products of interest such as protein, nucleic acids and polyesters like PHB.

### Status and significance of PIA-MVP

It is of interest to consider a physiological relationship between MV formation with PHB production. The artificial *E. coli* system lacks the native surface of carbonosomes as it can be found in PHB-producing native bacteria represented by *Ralstonia eutropha* or *Peudomonas putida*. Hence, the hydrophobic surface of the PHB produced in *E. coli* is at least partially exposed to the cytoplasm where it might come in contact with the cytoplasmic membrane, in particular because PHB granules in *E. coli* tend to localize close to the cell poles^16^. In case of a larger-cell-sized *Caryophanon latum*, PHB granules of Nile-red stained living cells were frequently found at or close to the cytoplasmic membrane at the early stages of PHB accumulation, as revealed by TEM^17^. After cell lysis, it was seen that PHB granules were in some frequent associated with MVs. Throughout the cell cycle, physical contact of PHB with the cytoplasmic membrane was observed from the view of the electron-translucent structures. These natural ecosystems would provide any insights into our artificial system PIA-MVP.

In this study, we present that the MV biogenesis took place in completely different principle in terms of the coupling with intracellular accumulation of PHB in native state of the wild-type strain with maintaining growth ability (Supplementary Fig. S2) and without any mutation. This should be a characteristic feature that is distinguishable from the other reported cases^7^. Also, PIA-MVP is a versatile platform methodology for efficient MV production that can be combined with the conventional strategies, genetical mutations^9,10^ and external addition of chemicals like glycine^7^ properly causing MV biogenesis. It might be feasible to substitute PHB with other type of biopolymers such as lactate-based polymer^18^. MVs are now attractive as a potential proteoliposomal carrier of vaccines from the viewpoints of the advantages of nano-sized admixtures, their immunological properties, and their structural stability^12^. In near future, PIA-MVP would contribute to utilization of MVs towards the wide range of applications.

## Conclusion

To date, many researchers have focused on the most-studied microbial polyester PHB accumulated in bacterial cells because it can be used as biodegradable bio-based plastic. Unexpectedly, we have discovered the biogenesis of MV in the PHB-producing recombinant *E. coli*. This discovery was achieved as a trigger of foam formation that many PHA researchers have often experienced during the microbial polymer fermentation. In such a sense, this study serves as a bridge between both research communities of PHB and MV, and will also reminds PHA researchers to carefully investigate a similar phenomenon from now on.

MV is also a well-known supramolecule that provides us with wide range of applications such as vaccine display, biofilm formation and drug delivery carrier. Here we propose a new “PIA-MVP” approach with presenting a mechanistic model for MV biogenesis. Our hypothetical model for PIA-MVP (illustrated in Fig. 7) is based on the finding that the internal pressure of PHB intracellular accumulation towards cell membrane architecture, probably leading to the MV formation as a result of envelope stress. The internal pressure can be accounted for by the two factors, production time-course of both supramolecules (Fig. 4) and morphological change (Fig. 6) of the cells caused by PHB accumulation in which effect is finely controlled by the concentration of glucose externally added as a carbon source (Fig. 5). This dose-dependency of PHB production by glucose concentration control straightly affects MV biogenesis and can be fine-tuned.

The microbial MV secretion system would function as a driving force for exo-vesiculation of any targets of interest. In near future, PIA-MVP will provide us with a versatile platform approach from both viewpoints of basic researchers executing the molecular mechanistic studies on membrane/peptidoglycan architecture, and wide-range applications such as delivery of their cargos to the target cells.

## Materials and methods

### Bacterial strains, plasmids and growth conditions

The strains and plasmids used in this study were listed (Table 2). *E. coli* BW25113 was used as host strain. The plasmid pGEM- *phaC*_*Re*_*AB*^6^ carrying PHA synthase gene (*phaC*_*Re*_ from *Ralstonia eutropha*), 3-ketothiolase gene (*phaA*_*Re*_ from *R. eutropha*), and acetoacetyl-CoA reductase gene (*phaB*_*Re*_ from *R. eutropha*), were used for PHB production.

**Table 2:** *Escherichia coli* strain and plasmid used in this study.

Strain or plasmid		Description	Reference
*Escherichia coli*			
BW	BW25113	*rrnB*T14 *ΔlacZ*WJ16 *hsdR*514 *ΔaraBAD*AH33	6
BW/PHB (+)	BW25113/ pGEM-*phaC*_Re_*AB*	BW25113 harboring pGEM-*phaC*_Re_*AB*	6
BW/PHB (−)	BW25113/pGEM-*phaC*_Re_(C319A)*AB*	BW25113 harboring pGEM-*phaC*_Re_(C319A)*AB*	6
*Plasmid*			
pGEM-*phaC*_Re_*AB*		pGEM vector carrying *phaC*_Re_, *phaA*_Re_ and *phaB*_Re_ genes	6
pGEM-*phaC*_Re_(C319A)*AB*		pGEM vector carrying *phaC*_Re*.*_ C319A mutant, *phaA*_Re_ and *phaB*_Re_ genes	6

The *E. coli* cells were usually cultured in LB medium (10 g/L Tryptone, 5 g/L Yeast extract, and 10 g/L NaCl) containing 2.0% (w/v) glucose for PHB production. When glucose-dependent PHB production level was examined, its concentration was varied as follows, 0, 0.25, 0.5, 1.0, 1.5, 2.0% (w/v). The culture medium for the strains harboring plasmid vector were supplemented with 50 mg/L ampicillin. All the test cultures were precultured in LB for 13 h at 30 °C and inoculated into 100 mL of fresh LB in a 500 mL Sakaguchi flask. The recombinant strains were cultivated with reciprocal shaking at 125 strokes/min.

### Electron microscopic observation

The culture supernatant of the recombinant strain of *E. coli* was subjected to electron microscopic observation. The cell pellets were collected by centrifugation (6000×*g*, 1 min) and fixed with 2.5% glutaraldehyde in 10 mM PBS at 4 °C for overnight. The fixed cells were washed three times with 10 mM PBS and then fixed additionally with 2% osmium oxide solution. The fixed cells were washed three times with water, dehydrated with ethanol, and then freeze dried with *t*-butyl alcohol. The freeze-dried cells were attached to carbon tape and sputter coated with osmium using an ion sputtering machine (HPC-1SW, Vacuum device). The cells were inspected using a scanning electron microscope under 5 kV (JSM-6700F, JEOL).

### Determination of cell size

The cell images were captured using a microscopy BZ-X700 (Keyence). The relative cell size was measured by flow cytometry using a SH800 cell sorter (SONY). The cells were harvested at 48 h and tenfold diluted sample with PBS was analyzed. The flow rate was set to 6 μL/min (pressure 1). Forward scatter data and back scatter data of 100,000 counts was recorded using SH800 software (SONY).

### Quantification of the total amount of PHB

After cultivation, the collected cells were washed three times with water and freeze-dried. The PHB contents in the cells was determined with determined with methanolized dried cell samples by gas chromatography (GC) as described previously^6^. Briefly, approximately 50 mg of dried cells were methanolized in 2 mL of 15:85% (v/v) sulfuric acid/methanol and 2 mL of chloroform at 100 °C for 140 min. After cooling the mixture, 1 mL of pure water was added, and the mixture was left until it separated into two layers. A portion of the chloroform layer was subjected to GC (GC-2030, Shimadzu) equipped with aflame ionization detector (FID), and methyl-esterified 3HB derived from P(3HB) was detected.

### MV isolation and quantification

MVs were isolated and quantified as described previously^8^. Briefly, 65 mL of culture supernatants (BW/PHB (+), BW/PHB (−) and BW) were filtered with a 0.45 μm pore size cellulose acetate filter (Merck Millipore), and ultracentrifuged for 1 h at 150,000×*g*, 4 °C. The pellets were resuspended in double distilled water for MV quantification. For further purification, MV pellets were resuspended in 45% iodixanol (Optiprep, AXIS-SHIELD) in HEPES-NaCl buffer and subjected to density gradient ultracentrifugation in an iodixanol gradient of 45–10%. For MV quantification, isolated MVs were dissolved in 200 μL of PBS were incubated with 1.25 μg/mL FM1-43 FX (Molecular Probes, Invitrogen) in phosphate-buffered saline (PBS). Then, MVs were measured at the excitation and emission wavelengths of 472 and 580 nm, respectively, using the microplate reader Synergy H1 (BioTek Instruments). The MV sample without staining by a FM1-43 FX or a FM1-43 FX alone was used as the negative controls. The particle size of MV was determined using NanoSight NTA 3.2 (Malvern Instruments). NanoSight particle tracking analysis was performed using isolated MVs. NanoSight particle analysis was performed in 60 s reads in triplicate, with the gain set to 10, detection threshold to 2 and camera level 13. Background from the corresponding blank samples was subtracted from each sample read and the average of the three reads was calculated and plotted as particle size versus number of particles per mL. For TEM observation, isolated MVs were stained with 2% uranyl acetate and inspected using a transmission electron microscope under 120 kV (JEM-2010, JEOL). Proteins involved in isolated MV were analyzed by sodium dodecyl sulfate-poly acrylamide gel electrophoresis (SDS-PAGE) with 12.0% polyacrylamide gels, and then stained with Coomassie Brilliant Blue (EzStain AQua, ATTA). The image was captured with ChemiDoc Imaging System (Bio-Rad). The full-length gel image is shown in Supplementary Fig. S3.

## Supplementary Information

Supplementary Information.

## Data Availability

All data generated or analyzed during this study are included in this published article.
