# Supplementary material for: Controllable secretion of multilayer vesicles driven by microbial polymer accumulation
Source: Sci Rep. 2022 Mar 1;12:3393. doi: 10.1038/s41598-022-07218-z (PMC8888611; doi:10.1038/s41598-022-07218-z)
Supplement: Supplementary file 1 — Supplementary Information. [file 41598_2022_7218_MOESM1_ESM.pdf]

Supplementary information for:

Controllable secretion of multilayer vesicles driven by microbial polymer accumulation

Sangho Koh[1,2], Michio Sato[3], Kota Yamashina[2], Yuki Usukura[4], Masanori Toyofuku[4,5,6], Nobuhiko Nomura[4,6] & Seiichi Taguchi[1,2*]

[1]*Graduate School of Science, Technology and Innovation, Kobe University, 1-1 Rokkodai-cho, Nada, Kobe 657-8501, Japan*

[2]*Department of Chemistry for Life Sciences and Agriculture, Faculty of Life Sciences and Agriculture, Tokyo University of Agriculture, 1-1-1 Sakuragaoka, Setagaya, Tokyo 156-8502, Japan*

[3]*School of Agriculture, Meiji University, 1-1-1 Higashimita, Tama, Kawasaki 214-8571, Japan*

[4]*Graduate School of Life and Environmental Sciences, University of Tsukuba, Tsukuba, Ibaraki 305-8572, Japan*

[5]*Suntory Rising Stars Encouragement Program in Life Sciences (SunRiSE), 8-1-1 Seikadai, Seika, Soraku, Kyoto, 619-0284, Japan*

[6]*Microbiology Research Center for Sustainability, University of Tsukuba, Ibaraki 305-8572, Japan*

*Correspondence:

Seiichi Taguchi (email: staguchi86@people.kobe-u.ac.jp)

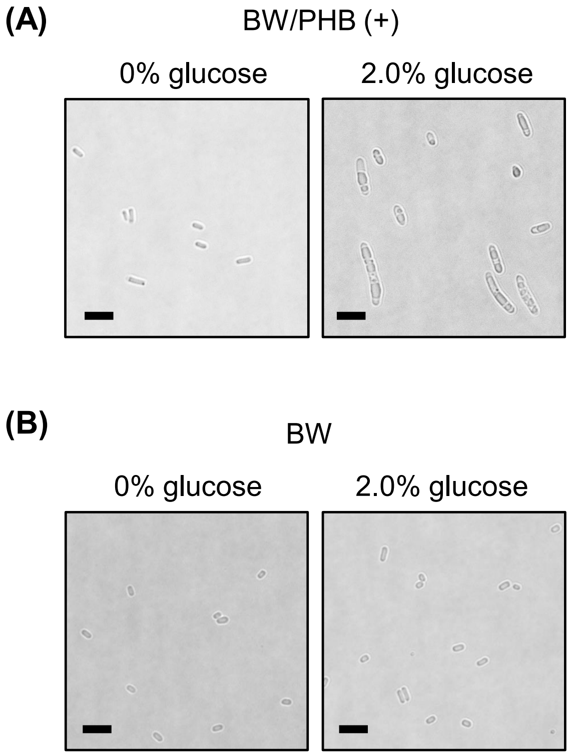

Supplementary Fig. S1 Morphological change of (A) BW/PHB (+) and (B) BW cells by varying glucose concentration.

Cells were cultivated in different glucose concentrations (0% and 2.0%) at 30°C for 48 h cultivation. As mentioned in the main text, significant morphological change was not observed for PHB-non producing BW strain at all.

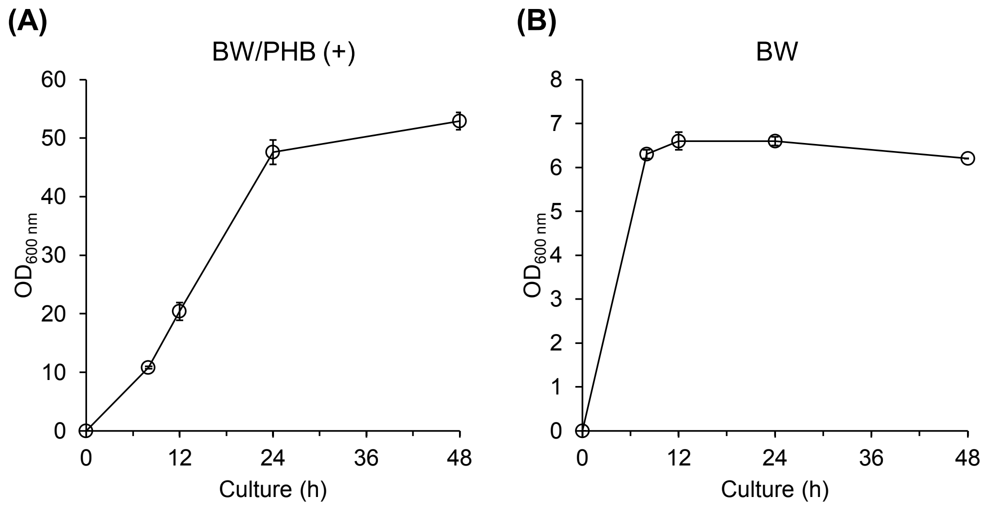

Supplementary Fig. S2 Growth curve of (A) BW/PHB (+) and (B) BW cells.
Cells were cultivated in in LB medium containing 2.0% glucose at 30°C for 48 h cultivation. Cell-lysis was not observed for BW/PHB (+) strain within 48 h cultivation.

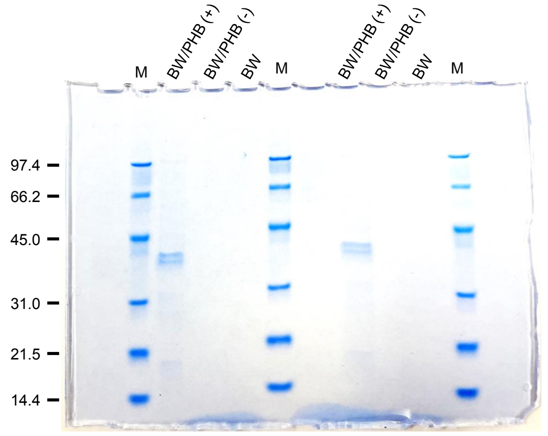

Supplementary Fig. S3 The full-length gel image of Fig. 3C.
